# A 3D adipogenesis platform to study the fate of fibro/adipogenic progenitors in muscular dystrophies

**DOI:** 10.1242/dmm.049915

**Published:** 2023-06-23

**Authors:** Alessio Reggio, Francesca De Paolis, Salma Bousselmi, Felice Cicciarelli, Sergio Bernardini, Alberto Rainer, Dror Seliktar, Stefano Testa, Carmine Cirillo, Paolo Grumati, Stefano Cannata, Claudia Fuoco, Cesare Gargioli

**Affiliations:** ^1^ Department of Biology, University of Rome ‘Tor Vergata’, 00133 Rome, Italy; ^2^ Cellular and Molecular Biology, Department of Biology, University of Rome ‘Tor Vergata’, 00133 Rome, Italy; ^3^ Department of Engineering, Università Campus Bio-Medico, 00128 Rome, Italy; ^4^ Institute of Nanotechnology (NANOTEC), National Research Council, 73100 Lecce, Italy; ^5^ Department of Biomedical Engineering, Techion Institute, 32000 Haifa, Israel; ^6^ Aix Marseille University, INSERM, Marseille Medical Genetics (MMG), 13005 Marseille, France; ^7^ Telethon Institute of Genetics and Medicine (TIGEM), 80078 Pozzuoli, Italy; ^8^ Department of Clinical Medicine and Surgery, Federico II University, 80138 Naples, Italy

**Keywords:** Muscular dystrophies, Adipogenesis, Fibro/adipogenic progenitors, β-catenin, LY2090314, Tissue engineering

## Abstract

In human dystrophies, progressive muscle wasting is exacerbated by ectopic deposition of fat and fibrous tissue originating from fibro/adipogenic progenitors (FAPs). In degenerating muscles, the ability of these cells to promote successful healing is attenuated, and FAPs aberrantly expand and differentiate into adipocytes and fibroblasts. Thus, arresting the fibro/adipogenic fate of FAPs, without affecting their physiological role, represents a valuable therapeutic strategy for patients affected by muscle diseases. Here, using a panel of adipose progenitor cells, including human-derived FAPs, coupled with pharmacological perturbations and proteome profiling, we report that LY2090314 interferes with a genuine adipogenic program acting as WNT surrogate for the stabilization of a competent β-catenin transcriptional complex. To predict the beneficial impact of LY2090314 in limiting ectopic deposition of fat in human muscles, we combined a poly-ethylene-glycol-fibrinogen biomimetic matrix with these progenitor cells to create a miniaturized 3D model of adipogenesis. Using this scalable system, we demonstrated that a two-digit nanomolar dose of this compound effectively represses adipogenesis at higher 3D scale, thus indicating the potential for LY2090314 to limit FAP-derived fat infiltrates in dystrophic muscles.

## Teaser

**Summary:** LY2090314 suppresses 2D and 3D adipogenesis of fibro/adipogenic progenitors by stabilizing a transcriptional-competent β-catenin complex.

## INTRODUCTION

Fibro/adipogenic progenitors (FAPs) are interstitial cells residing in all tissues of mesenchymal origin. Independently from the source, these cells share conserved roles and functions by being primarily devoted to the overall maintenance of tissue homeostasis (Contreras et al., 2021; Giuliani et al., 2022). In skeletal muscle tissue, FAPs contribute to sustain the regenerative potential and mass of postnatal muscles by preserving and supporting adult muscle stem cells, namely satellite cells (Uezumi et al., 2021; Wosczyna et al., 2019). However, in degenerating muscles, the ability of FAPs to promote successful healing is attenuated, and these cells tend to differentiate into adipocytes and fibroblasts, further aggravating degeneration (Madaro et al., 2018; Santini et al., 2020; Uezumi et al., 2011). Ectopic deposition of adipocytes exacerbates muscular dystrophies (Emery, 2002), including Duchenne muscular dystrophy, limb girdle muscular dystrophies, Emery-Dreyfuss muscular dystrophy and other myopathies (Bosnakovski et al., 2017; Hogarth et al., 2019; Uezumi et al., 2011, 2014). Adipose infiltrates are also common in obesity (Akhmedov and Berdeaux, 2013; Buras et al., 2019; Pérez-Díaz et al., 2022) and in type II diabetes (Farup et al., 2021), and correlate with muscle wasting during aging (Volpi et al., 2004).

Pharmacological agents capable of altering FAP adipogenesis have been identified (Cordani et al., 2014; Farup et al., 2021; Kasai et al., 2017; Kopinke et al., 2017; Mozzetta et al., 2013; Reggio et al., 2019, 2020b), concretizing only recently the possibility to significantly mitigate intramuscular fatty degeneration. Recently, endogenous WNT ligands emerged as a critical regulator of FAP behavior, suggesting that the manipulation of WNT pathway components, via small molecules, may represent a feasible strategy for limiting the adipogenic drift of these cells (Reggio et al., 2020b). In the past years, our group uncovered GSK3 as a transducer that controls adipogenesis, while identifying GSK3 inhibitors as valuable candidates to impair FAP adipogenic fate (Reggio et al., 2020b). Specifically, we demonstrated that short-term exposure to LY2090314, a highly potent GSK3 inhibitor, is capable of limiting fatty degeneration in glycerol-degenerating muscles as well as of inhibiting adipogenesis of FAPs extracted from dystrophic *mdx* muscles (Reggio et al., 2020b).

In the present work, we provide pharmacological evidence that an active β-catenin transcriptional complex intimately represses the adipogenic program of adipose progenitor cells, including human FAPs (hFAPs). Using liquid chromatography–tandem mass spectrometry (LC-MS/MS) profiling, we demonstrated that LY2090314 exerts WNT-mimicking properties and inhibits hFAP adipogenesis through, at least, two independent mechanisms, both mediated by β-catenin. A primary mechanism is ascribed to the role of LY2090314 in inhibiting GSK, while a second indirect mechanism corroborates GSK blockage via WNT5A upregulation.

To demonstrate the clinical value of LY2090314, we established a novel method to study fat infiltrates in three-dimensional (3D) scale. Biomimetic matrices are paving the way for the generation of affordable humanized models for medical and research applications, allowing the scale up of cell-based evidence in more complex and clinically valuable tissue-like systems (Celikkin et al., 2021; Fernández-Garibay et al., 2022; Fuoco et al., 2012). Using a photopolymerizable poly-ethylene-glycol-fibrinogen (PF) hybrid biomimetic matrix with FAPs, we generated a miniaturized fatty infiltrate 3D model. Employing this novel platform, we showed that a two-digit nanomolar dose of LY2090314 is effective at repressing 3D adipogenesis, thus providing proof of principle for the use of this compound to limit FAP-derived fat infiltrates in diseased muscles. In addition, our miniaturized 3D fatty depot represents a scalable system to collect cellular and molecular details regarding the impact of drugs while predicting, in a 3D scenario, clinical outcome, unwanted side effects and complementary molecular targets for combinatorial anti-adipogenic therapies.

## RESULTS

### A transcriptional-competent β-catenin complex is the most downstream effector repressing adipogenesis

We performed initial experiments on 3T3-L1 cells, which are immortalized murine preadipocyte cells widely used to shed light on the molecular routes that drive the transition of preadipocytes into lipid-laden cells (Rosen and Spiegelman, 2000). Adipogenesis of this cell line is promoted by supplementation of the adipocyte differentiation medium (ADM) with pro-differentiation hormones: insulin, synthetic glucocorticoids, such as dexamethasone, and the phosphodiesterase inhibitor 1-methyl-3-isobutyl xanthine (IBMX) (Rosen and Spiegelman, 2000).

After 3[Supplementary-material sup1]days of ADM culture, 3T3-L1 cells started to express canonical adipogenic markers as a sign of the activated adipogenic differentiation. Specifically, the expression of two members of the Cebp family (*Cebpa* and *Cebpb*) as well as the master adipogenic gene *Pparg* were significantly upregulated by ADM exposure (Fig. S1A). Incubation with ADM upregulated the expression of PPARγ, which exclusively localizes in the nucleus of cells undergoing adipogenesis (Fig. S1B). A time-course experiment confirmed the differentiation kinetics of these cells (Fig. S1C). Specifically, PPARγ expression (used as an early adipogenic marker) was detected on the third day after ADM incubation, while perilipin-1 (which marks terminally differentiated cells) started to appear on the fifth day and progressively increased until complete maturation of the 3T3-L1 cells (Fig. S1C). The phenotype conversion into adipocytes can be revealed using a highly specific dye, such as Oil Red O (ORO), highlighting mature lipid-laden cells (Fig. S1D,E). Therefore, the presented results demonstrated that 3T3-L1 cells do not spontaneously differentiate into adipocytes. Conversely, ADM incubation triggers a well-defined kinetics leading to the conversion of these progenitors into mature lipid-laden cells.

**Fig. 1.**
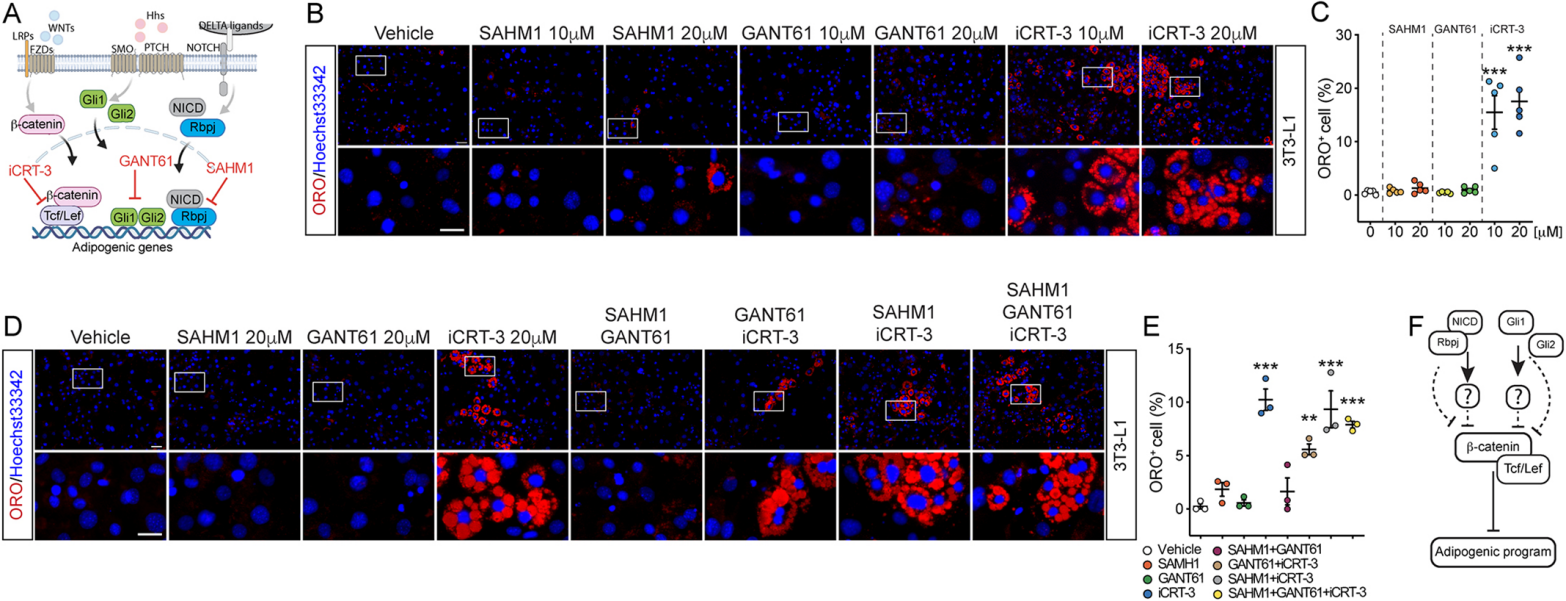
**A functional β-catenin transcriptional complex suppresses adipogenesis of 3T3-L1 cells.** (A) Schematic representation of the drug perturbation experimental plan. (B) Representative immunofluorescence analysis showing Oil Red O (ORO) staining after the drug perturbation experiment described in A. Cells were treated with the reported compounds for 72 h in growth medium and then cultured for six additional days in a compound-free medium. Nuclei (blue) were revealed using Hoechst 33342. (C) Dot plot reporting the fraction of ORO-positive cells in the experiment in B. (D) Representative immunofluorescence analysis showing ORO staining after the drug perturbation experiments with different pairwise combinations. (E) Dot plot reporting the fraction of ORO-positive cells in the experiment in D. (F) Schematic model summarizing the results presented in this figure. All micrographs were captured at 20× magnification. Scale bars: 50 μm; 25 μm (insets). Statistical significance was determined by one-way ANOVA. Images and data are representative of at least three independent biological repeats. All data are presented as mean±s.e.m. **P<0.01; ***P<0.001.

Embryonic pathways such as NOTCH, SHH and WNT/β-catenin control several aspects of adipose biology, including embryogenesis, postnatal development, induction of differentiation and homeostasis (Rosen and MacDougald, 2006). Even though this notion has been well consolidated in the literature, our knowledge mostly comes from experiments that consider one of these pathways at a time. Thus, the relevance of one of these pathways compared to the others is not yet well understood. For these reasons, we sought to identify which signaling pathway, among NOTCH, SHH and WNT/β-catenin, has a prominent role in repressing the adipogenic programs of 3T3-L1 cells. We selected SAHM1, GANT61 and iCRT-3 to block, with high selectivity, the transcriptional program of NOTCH, SHH and WNT/β-catenin, respectively (Fig. 1A). Briefly, SAHM1 disrupts binding between the NOTCH intracellular domain (NCID) and RBPJ, blocking the activation of NOTCH target genes. Similarly, iCRT-3 displaces the interaction between β-catenin and its transcriptional partner TCF/LEFs. By contrast, GANT61 blocks GLI1 and GLI2 activity by inhibiting SHH-mediated signaling (Fig. 1A). Such an approach offers the advantage of targeting, as downstream as possible, such signaling routes, with reduced interference of upstream and collateral pathways.

We tested the inhibitory effect of these small molecules in unstimulated 3T3-L1 cells, with the aim of identifying whether a single inhibition pathway stimulates adipogenesis in the absence of the ADM (Fig. 1B). Each compound was tested at two doses (10 and 20 µM), in agreement with literature reports demonstrating the safety and non-cytotoxicity of these compounds at these concentrations (Ashley et al., 2015; Mazumdar et al., 2011; Sharma et al., 2017). We found that only the inhibition of the transcriptional activity of β-catenin by iCRT-3 was sufficient to stimulate adipogenesis in cultured 3T3-L1 cells in the absence of adipogenic stimuli (Fig. 1B), as revealed by the fractions of ORO-positive cells (Fig. 1C). This suggests that the transcriptional activity of β-catenin is solely responsible for repressing adipogenesis of 3T3-L1 preadipocytes. Combinatorial treatments of these compounds, tested at 20 µM, clearly demonstrated that adipogenesis only takes place in the presence of iCRT-3 (Fig. 1D,E), suggesting that β-catenin is the most downstream effector repressing adipogenesis. Hence, the control of NOTCH and SHH on this phenotype is either dependent on β-catenin or dependent on an intermediate factor that, in turn, impairs β-catenin transcriptional activity (Fig. 1F).

### LY2090314 raises β-catenin concentrations and abrogates adipogenesis of 3T3-L1 cells and hASCs

The data collected revealed β-catenin as a molecular determinant controlling the adipogenic differentiation of 3T3-L1 cells. Thus, we sought to investigate β-catenin dynamics during 3T3-L1 cell adipogenesis. To this end, we monitored the levels of β-catenin protein during the first 3 days of 3T3-L1 cell differentiation. Notably, western blot analysis revealed a progressive temporal decrease in β-catenin expression during 3T3-L1 cell adipogenesis (Fig. S2A). Immunofluorescence analysis displayed a significant reduction in the β-catenin signal throughout the cellular compartment of the cells undergoing adipogenesis, especially in the nucleus (Fig. S2B,C). To verify these observations, from a biochemical point of view, we processed whole-cell lysates to enrich cytoplasm and nuclear protein fractions. Three days of incubation with the ADM were sufficient to cause almost total depletion of β-catenin from the nuclear compartment, confirming the reliability of our microscopy data (Fig. S2D).

To clarify the molecular event(s) controlling the intracellular levels of β-catenin, we performed a pharmacological-based experiment to monitor β-catenin levels upon early ADM stimulation in the presence of cycloheximide, MG132 and concanamycin A, compounds that block translation, proteosome and autophagy activities, respectively. Intriguingly, only MG132 was effective at increasing β-catenin concentrations, suggesting that β-catenin is subjected to a proteasome-dependent degradation (already at 24 h) after ADM exposure (Fig. S2E,F). MG132 exposure promoted the accumulation of a small band of higher molecular mass corresponding to the monoubiquinated form of β-catenin (Fig. S2E), an event that was consistently lost when translation was inhibited by cycloheximide. Overall, our data demonstrated that the activation of the adipogenic program requires a decrease in the cellular concentrations of β-catenin, especially its nuclear fraction, and that such reduction is mediated by proteasome degradative processes.

**Fig. 2.**
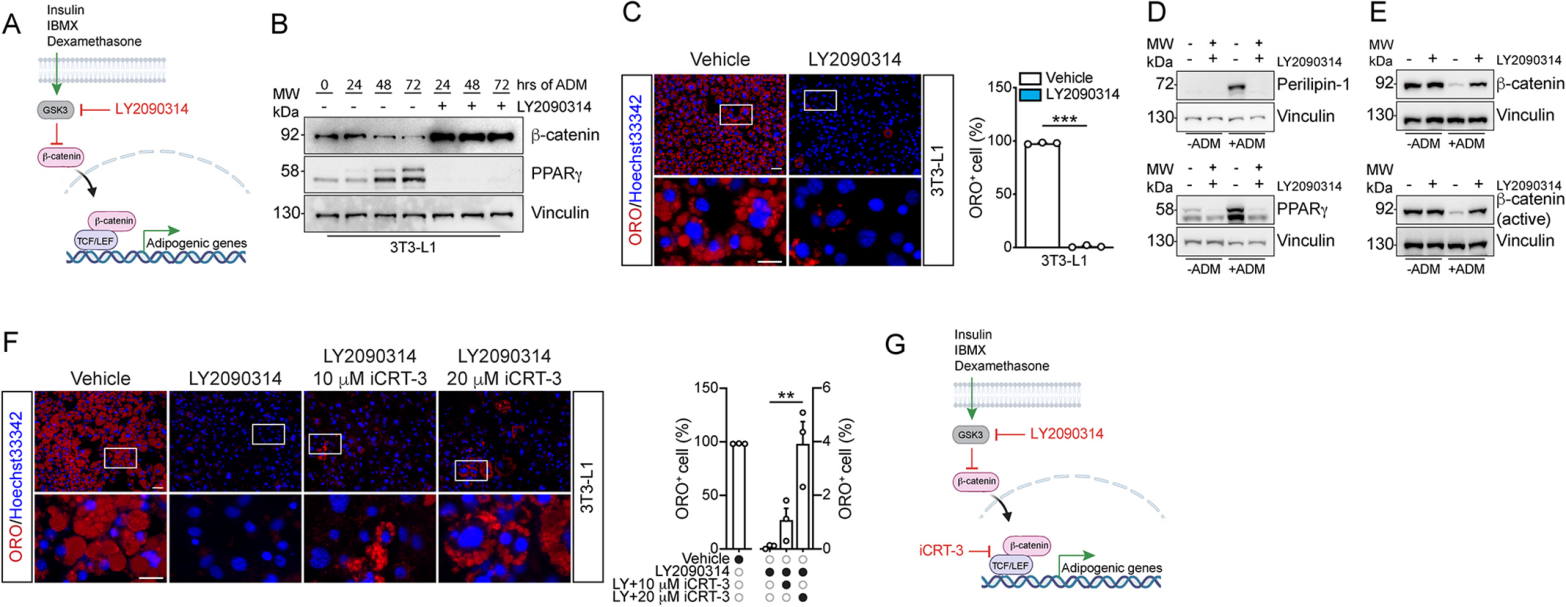
**LY2090314 stabilizes β-catenin intracellular amount and represses adipogenesis of 3T3-L1 cells.** (A) Schematic model of action of LY2090314. IBMX, 1-methyl-3-isobutyl xanthine. (B) Representative western blot showing the expression of β-catenin and PPARγ proteins during the first 3 days of differentiation of 3T3-L1 cells. Vinculin was used as loading control. (C) Representative immunofluorescence analysis showing differentiated 3T3-L1 cells in the presence/absence of 20 nM LY2090314 (left). Cells were cultured for 3 days in adipocyte differentiation medium (ADM) in the presence/absence of LY2090314 and then cultured for six additional days in a compound-free maintenance medium. Nuclei (blue) were revealed using Hoechst 33342. The bar plot (right) reports the percentage of ORO-positive cells (red) in both conditions. (D) Representative western blots showing the expression of perilipin-1 and PPARγ proteins at the end point of the differentiation program of 3T3-L1 cells, with or without exposure to 20 nM LY2090314. Vinculin was used as loading control. (E) Representative western blots showing the expression of β-catenin and non-phospho active β-catenin proteins at the end point of the differentiation program of 3T3-L1 cells, with or without exposure to 20 nM LY2090314. Vinculin was used as loading control. (F) Representative immunofluorescence analysis showing differentiated 3T3-L1 cells in the presence/absence of a combinatorial treatment of 20 nM LY2090314 and 10 or 20 µM iCRT-3 (left). Nuclei (blue) were revealed using Hoechst 33342. The bar plot (right) reports the percentage of ORO-positive cells (red) in all the considered conditions. (G) Schematic model summarizing mechanistic details of LY2090314 and iCRT-3 in the adipogenic pathway. All micrographs were captured at 20× magnification. Scale bars: 50 μm; 25 μm (insets). Statistical significance was determined by paired two-tailed Student’s *t*-test (C) and one-way ANOVA (F). Figures and data are representative of at least three independent biological repeats. All data are presented as mean±s.e.m. **P<0.01; ***P<0.001.

To assess whether interfering with β-catenin levels, namely decreasing them, has an impact on the adipogenic fate of 3T3-L1 cells, we focused our attention on GSK3, which is known to prime β-catenin for its degradation via phosphorylation. To test this hypothesis, we used LY2090314 to stimulate β-catenin accumulation within cells (Fig. 2A). LY2090314 is a highly selective GSK3 inhibitor capable of exerting its action in a nanomolar concentration range (Atkinson et al., 2015; Kunnimalaiyaan et al., 2018). Short-term incubation (3 days) with 20 nM LY2090314 was sufficient to prevent β-catenin degradation while increasing the amount of β-catenin within 3T3-L1 cells undergoing adipogenesis (Fig. 2B). Moreover, we found that such stabilization paralleled with the absence of PPARγ expression, making these cells incapable of engaging the adipogenic program. Consistently, 3T3-L1 cells failed to differentiate into mature adipocytes upon LY2090314 exposure, as revealed by ORO staining (Fig. 2C) and by the expression of perilipin-1 and PPARγ at the end point of the differentiation program (Fig. 2D). Remarkably, short-term exposure to LY2090314 was sufficient to sustain the total β-catenin, as well as the non-phospho active form (i.e. nuclear) of β-catenin, in these cells, thus promoting β-catenin accumulation within the nucleus (Fig. 2E).

To corroborate such data in a primary adipose progenitor cell model, we collected primary human adipose stromal cells (hASCs), a primary cell suspension that is enriched in adipose progenitors capable of differentiating into primary adipocytes. Primary hASCs were cultured and differentiated using standard protocols, giving rise to highly differentiated ORO-positive adipocytes (Fig. S2G). β-catenin was demonstrated to be subjected to the same lowering kinetics in differentiating hASCs as in 3T3-L1 cells (Fig. S2H), suggesting that β-catenin degradation hallmarks a genuine adipogenic program. Incubation with 20 nM LY2090314 was sufficient to prevent β-catenin degradation while significantly increasing the intracellular concentrations of this protein effector (Fig. S2I). Such an event parallels the failure of hASCs to acquire a terminally differentiated phenotype, as revealed by the absence of ORO-positive adipocytes (Fig. S2J). Expression of PPARγ and perilipin-1 (Fig. S2K) was downregulated in hASCs exposed to anti-adipogenic doses of LY2090314 compared to that in hASCs without LY2090314 exposure.

To mechanistically prove that LY2090314 is acting by stabilizing a nuclear transcriptionally active complex containing β-catenin, we attempted to rescue adipogenesis in LY2090314-treated 3T3-L1 cells by co-exposing cells to increasing doses of iCRT-3. Incubation with iCRT-3 significantly re-sensitized these cells to the ADM, leading to the formation of mature adipocytes. Therefore, GSK blockage by LY2090314 increases β-catenin intracellular levels and inhibits adipogenesis by stabilizing the active β-catenin transcriptional complex (Fig. 2F).

Altogether, these data indicate that adipogenesis of adipose progenitors can be modulated by altering β-catenin concentrations and that LY2090314 is highly effective at altering the differentiation fate of cell models with adipogenic potential (Fig. 2G).

### LY2090314 impairs adipogenesis of primary FAPs of murine and human origin

Alteration of FAP homeostasis is commonly found in muscle degenerative disorders. In muscular dystrophies, myofiber breakdown coupled with inefficient muscle regeneration leads to the progressive replacement of the contractile tissue with fat, which largely infiltrates muscle fibers. The cell origin of this non-muscle tissue has been ascribed to the altered behavior of FAPs (Giuliani et al., 2022; Theret et al., 2021). In a diseased or massively injured environment, as in the case of glycerol damage, FAPs lose their pro-regenerative role and, in this context, fully experience their mesenchymal nature by differentiating into adipocytes (Stumm et al., 2018), as highlighted by the tight relationship between PDGFRα (the FAP-distinctive marker) positivity and perilipin-1 depots (Fig. S3A). For this reason, FAPs are considered a good cell target for developing novel therapies aimed at limiting fatty degenerative diseases in muscular dystrophies (Kopinke et al., 2017; Reggio et al., 2020a,b; Zhao et al., 2020). Consistently, we previously demonstrated that, once isolated from *mdx* dystrophic mice, FAPs are prone to adipogenesis and that short-term exposure to LY2090314 limits this fate (Reggio et al., 2020b). To confirm these findings in a more robust setup, we isolated wild-type murine FAPs (mFAPs) from three different C57/BL6J mice and subjected these mFAP preparations to adipogenic differentiation (Fig. S3B). Adipogenic induction stimulated the expression of PPARγ while downregulating the expression of β-catenin (Fig. S3C). By contrast, 20 nM LY2090314 sustained β-catenin levels and downregulated PPARγ expression (Fig. S3C). Consistently, iCRT-3 increased the fraction of mFAP-derived adipocytes in a dose-dependent manner (Fig. S3D), by inhibiting active β-catenin transcriptional complexes. Therefore, mFAP-derived adipocytes were not present in cell cultures exposed to LY2090314 compound (Fig. S3E). Collectively, these data indicate LY2090314 as a candidate agent to counteract fat infiltrates in muscular dystrophies, by reducing FAP adipogenic propensity.

To formally prove the biological relevance of our model and clinical impact of this pharmacological agent, we decided to explore the effect of LY2090314 compound on hFAPs. To collect multidimensional information from human-derived samples, we decided to couple standard differentiation assays with state-of-the-art mass spectrometry (MS)-based proteomics, allowing the dissection of molecular details from hFAP cultures, at high resolution and unbiased level.

**Fig. 3.**
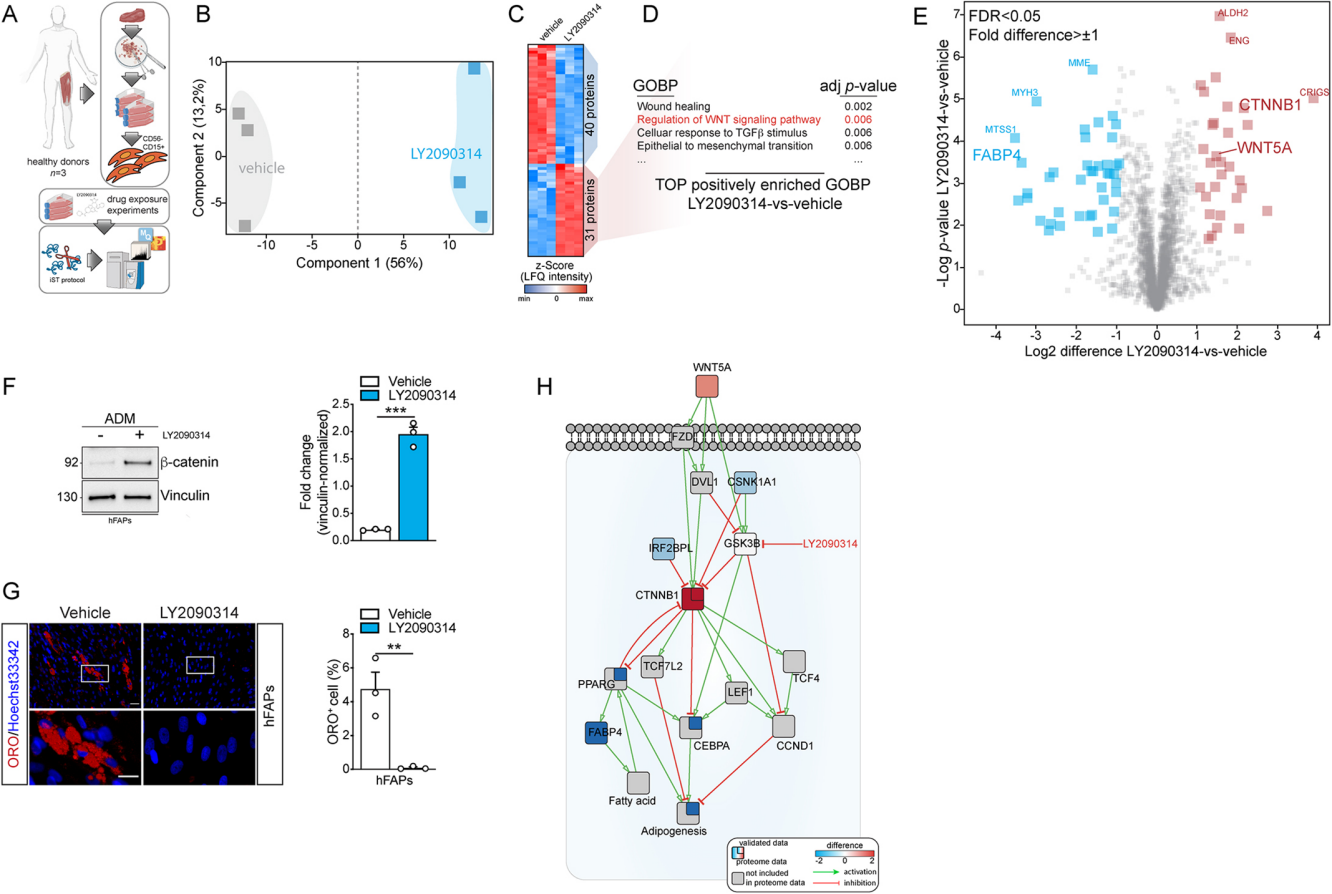
**LY2090314 abrogates adipogenesis of murine and human fibro/adipogenic progenitors (FAPs).** (A) Representative scheme showing the collection and utilization of human-derived FAPs (hFAPs). iST, in-StageTip. (B) Principal component analysis showing sample segregation of hFAP proteomes upon exposure to LY2090314 or vehicle. (C) Heatmap showing significantly [false discovery rate (FDR)<0.05; fold difference>±1] regulated proteins in the LY2090314-versus-vehicle comparison. LFQ, label-free quantitation. (D) Gene Ontology terms that are positively enriched (adjusted *P*<0.01) in the LY2090314-versus-vehicle comparison. GOBP, Gene Ontology biological process. (E) Volcano plot showing positively and negatively enriched proteins in the LY2090314-versus-vehicle comparison. (F) Representative western blot showing the expression of β-catenin in hFAPs in the absence/presence of 20 nM LY2090314 (left). The bar plot (right) reports the densitometric values of β-catenin in both conditions. (G) Representative immunofluorescence analysis showing differentiated hFAPs in the presence/absence of 20 nM LY2090314 (left). Cells were cultured for 3 days in ADM in the presence/absence of LY2090314 and then cultured for six additional days in a compound-free maintenance medium. Nuclei (blue) were revealed using Hoechst 33342. The bar plot (right) reports the percentage of ORO-positive cells in both conditions. (H) Network reporting the mode of action of LY2090314 in repressing the adipogenic program of hFAPs. Statistical significance was determined by paired two-tailed Student’s *t*-test. Images and data are representative of at least three independent biological repeats. All data are presented as mean±s.e.m. **P<0.01; ***P<0.001.

To this end, we purified FAPs from human skeletal muscle biopsies of three healthy donors to establish primary cultures of CD56^−^ (NCAM1^−^)/CD15^+^ progenitors (Arrighi et al., 2015; Suárez-Calvet et al., 2021), referred to as hFAPs (Fig. 3A). Next, for each donor, we exposed the resulting primary cultures to ADM, either supplemented with the vehicle or with 20 nM LY2090314, for 3 days. Upon 3 days of adipogenic priming, hFAPs were harvested for proteomics (Fig. 3A). An in-solution LC-MS/MS quantitation approach allowed us to identify ∼3300 unique proteins (Table S1). Principal component analysis revealed that the collected proteomes efficiently discriminate different samples, grouping them according to their treatment conditions (i.e. vehicle or LY2090314) (Fig. 3B). LY2090314 affects the proteome of FAPs differentiating into adipocytes: ∼3% of the FAP proteome was found to be significantly different [false discovery rate (FDR)<0.05, fold difference>±1], allowing the identification of 71 differentially regulated proteins (31 upregulated and 40 downregulated) (Fig. 3C).

Next, we interrogated Gene Ontology (GO) libraries to unbiasedly identify biological terms that are positively (Table S2) or negatively (Table S3) enriched by LY2090314 compound. According to their adjusted *P*-value, WNT-related terms were found to be over-represented in cultures exposed to LY2090314 (Fig. 3D; full enrichment analysis is provided in Table S2), implying that the anti-adipogenic properties of this compound may be ascribed to its ability to act as a WNT-phenocopying agent. As shown in the volcano plot representation in Fig. 3E, β-catenin (CTNNB1) protein was significantly and massively induced by LY2090314, whereas the adipogenic marker FABP4 was among the most depleted proteins. Consistently, short-term incubation with 20 nM LY2090314 sustained β-catenin expression and completely abrogated the ability of hFAPs to differentiate into adipocytes in the presence of ADM (Fig. 3F,G). Therefore, LY2090314 represses adipogenesis by acting as a WNT surrogate in hFAPs.

Surprisingly, we found that LY2090314 also increased the expression of WNT5A (Fig. 3E), which we previously demonstrated to be capable, via a β-catenin-dependent mechanism*,* of restricting adipogenesis in mFAPs (Reggio et al., 2020b).

To summarize the results from our proteomic survey, we mapped the 71 significantly modulated proteins (input nodes) onto a literature-derived network of signaling and physical interactions. For this purpose, we used the SIGNOR database through a recently developed application in the Cytoscape environment. To increase the connectivity between our protein entities, we used the possibility to include ‘bridge’ proteins via a native SIGNOR algorithm (Lo Surdo et al., 2023).

The resulting network encompasses 51 nodes, including receptors, signal transducers, transcription factors and phenotypes; relationships between nodes are either positive (activations) or negative (inhibitions) (Table S4). Here, we were capable of identifying a network module centered on β-catenin (Fig. S3F). Moreover, to increase the coverage of information, we also included the expression values for those protein entities that were dosed/quantified throughout this study (Fig. 3H). The subnetwork accurately describes incoming and outgoing stimuli of β-catenin while highlighting its inhibitory effects on adipogenic transcription factors, especially PPARγ and CEBPA. Such inhibitory effects are potentiated by the upregulation of WNT5A, which stabilizes β-catenin by blocking GSK3 (Fig. 3H).

Altogether, these data demonstrated that the anti-adipogenic role of LY2090314 is ultimately mediated by β-catenin through, at least, two independent mechanisms: a primary mechanism that is ascribed to the role of LY2090314 in inhibiting GSK kinase, and a second indirect mechanism that corroborates GSK blockage via WNT5A.

### Development of a 3D adipogenic model to test LY2090314 efficacy in mitigating fatty degenerative diseases

**Fig. 4.**
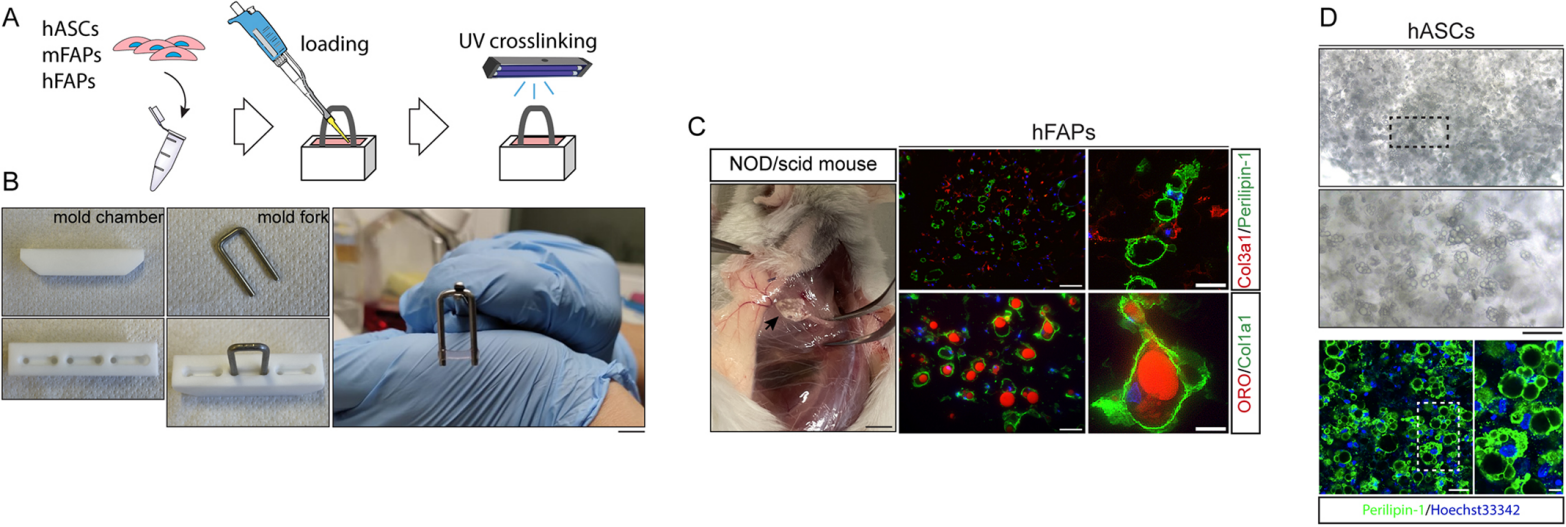
**LY2090314 suppresses 3D adipogenesis of murine and human FAPs.** (A) Representative scheme showing the generation of a miniaturized 3D model of fat infiltration using poly-ethylene-glycol-fibrinogen (PF) hydrogel. hASC, human adipose stromal cell; hFAP, human FAP; mFAP, murine FAP. (B) Components (i.e. the mold chamber and fork) for the generation of a 3D model of fat infiltration using PF hydrogel. A representative polymerized PF construct is also shown. Scale bar: 1 cm. (C) A previously transplanted PF construct containing hFAPs was explanted after 30 days (left). Scale bar: 1 cm. Representative cryosections of the explanted construct showing hFAPs expressing COL3A1 (red) and perilipin-1 (green) (right, top row), and COL1A1 (green) and ORO (red) (right, bottom row). Scale bars: 50 μm; 25 μm (insets). (D) PF hydrogel construct loaded with hASCs induced to differentiate into adipocytes using standard protocols. 3D differentiated adipocytes with evident lipid droplets are evident in brightfield microscopy (top). Scale bar: 100 µm; inset is an enlarged view. Representative whole-mount immunofluorescence showing perilipin-1 expression (green) in differentiated hASC-derived adipocytes (bottom). Scale bars: 50 µm; 10 µm (inset).

In our laboratory, we developed and optimized reliable methods for the generation of 3D organoids using muscle progenitor cells (Fuoco et al., 2015). The system is based on a photopolymerizable mixture of poly-ethylene-glycol and fibrinogen (PF) that was extensively demonstrated as (1) biocompatible and U.S. Food and Drug Administration (FDA) approved, (2) suitable for 3D culture of myogenic progenitor cells (Testa et al., 2020), and (3) adapted for the generation of mature and functional human and murine myosubstitutes that efficiently graft in host recipient muscles (Costantini et al., 2021). Starting from these notions, we tested the possibility of generating 3D models of adipogenesis by culturing adipose progenitors in a PF-based 3D microenvironment. To this end, hFAPs were resuspended in a 8 mg/ml PF matrix scaffold (Fig. 4A). The resulting mixture was then loaded in a polytetrafluoroethylene (PTFE) caster and polymerized by exposing the mold chamber to non-toxic and low-penetrating UV light (365 nm) (Fig. 4A,B). The polymerized PF 3D construct was stable, anchored to the mold fork, had a volume of ∼1 cm^3^ and was easily manipulable in classical culture vessels (Fig. 4B). Suprascapular transplantation of hFAP organoids in immunodeficient mice resulted in vascularized fibro/adipose depots (Fig. 4C), resembling those that can be found in some human fatty degenerative conditions. As a consequence of a spontaneous fibro/adipose differentiation drift, cryosections of these organoids showed the presence of hFAPs that express myofibroblast and adipocyte markers such as Collagens and perilipin-1, respectively (Fig. 4C).

Moreover, we generated surrogate models of fat pad by using hASCs. Indeed, in this 3D culture, state hASCs maintained the ability to respond to the ADM and differentiated into mature adipocytes with huge perilipin-1-positive lipid droplets (Fig. 4D), suggesting the suitability of this system to generate 3D models of fat infiltrates.

### LY2090314 inhibits 3D adipogenesis of mFAPs and hFAPs

**Fig. 5.**
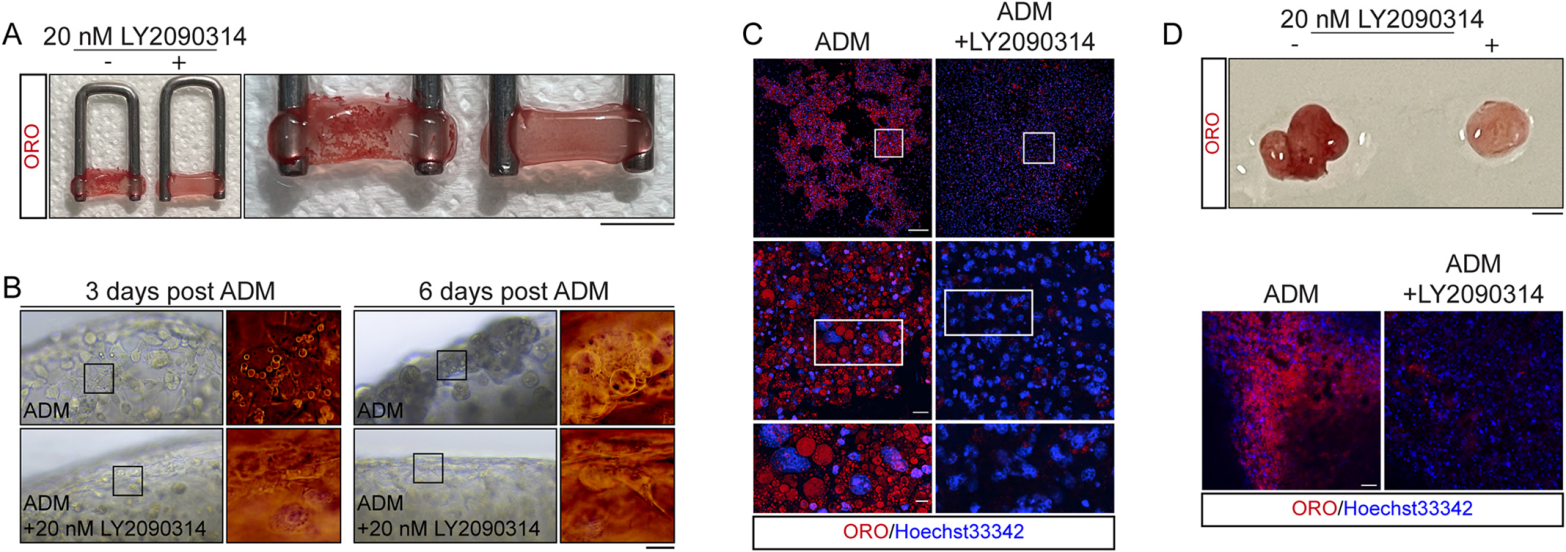
**LY2090314 inhibits 3D adipogenesis of mFAPs and hFAPs.** (A) Representative ORO staining of PF constructs previously loaded with mFAPs. Both constructs were exposed to ADM in the absence/presence of 20 nM LY2090314. Scale bar: 1 cm. (B) Representative brightfield micrographs showing mFAPs undergoing adipogenesis in the absence/presence of 20 nM LY2090314, at 3 and 6 days after ADM exposure. Scale bar: 15 µm. (C) Representative whole-mount immunofluorescence showing ORO labeling in mFAP-derived adipocytes (red) differentiated in the absence/presence of 20 nM LY2090314. Scale bars: 100 µm (top); 50 µm (middle); 10 µm (bottom). (D) Representative ORO staining of PF constructs previously loaded with hFAPs (top). Scale bar: 1 cm. Representative whole-mount immunofluorescence showing ORO labeling in hFAP-derived adipocytes (red) differentiated in the absence/presence of 20 nM LY2090314 (bottom). Scale bar: 50 µm. Images and data are representative of at least three independent biological repeats.

To this end, we tested the ability of LY2090314, at a concentration of 20 nM, to inhibit 3D mFAP/hFAP adipogenesis. In the absence of the GSK3 inhibitor, mFAPs differentiated into adipocytes, as revealed by the ORO-positive area visible throughout the 3D construct (Fig. 5A). By contrast, incubation with 20 nM LY2090314 prevented the formation of such visible ORO-positive area (Fig. 5A). The longitudinal microscopy-based monitoring of these constructs clarified that LY2090314 prevented the accumulation of lipid droplets in these 3D models of fatty infiltrates (Fig. 5B), as previously observed for those cells cultured in standard 2D culture vessels. Whole-mount confocal microscopy demonstrated that highly mature mFAP-derived adipocytes were only observed in the absence of LY2090314 (Fig. 5C). Consistently, hFAP 3D constructs failed to form highly differentiated 3D fat depots when exposed to 20 nM LY2090314 (Fig. 5D). The overall data presented in this study demonstrated that a two-digit nanomolar dose of LY2090314 compound is effective at inhibiting adipogenesis in a complex 3D environment, thus offering rational proof for engaging valuable studies testing clinical applications of LY2090314.

## DISCUSSION

Human muscular dystrophies are further worsened by intramuscular infiltrations of ectopic tissues, including fat, scar and, occasionally, mineralized tissue. As an aggravating factor, adipose infiltrates hinder the supply of nutrients to muscle fibers, limit muscle regeneration and exacerbate muscle deterioration. Non-physiological adipocytes also infiltrate the skeletal muscles of individuals with sarcopenia, or those with obesity and type II diabetes, contributing to the morbidity.

The origin of this deleterious fat is now ascribed to the altered differentiation behavior of FAPs (Uezumi et al., 2011), a cell population that resides in the interstitial and perivascular space of the skeletal musculature (Joe et al., 2010; Santini et al., 2020). In the past 10 years, several groups have specialized their research in the growing field of FAP biology, elucidating roles and molecular mechanisms that helped to rationalize FAP physiology and pathogenesis, while providing concrete tools for halting or limiting their detrimental effects in muscle diseases (Giuliani et al., 2022; Hogarth et al., 2019).

Hedgehog and NOTCH pathways have been implicated in controlling the adipogenic fate of FAPs, and alterations to their signaling cascades have been identified in FAPs from diseased muscles (Kopinke et al., 2017; Marinkovic et al., 2019). In recent years, muscle-derived WNT ligands have emerged as critical niche factors capable of modulating FAP fate (Fu et al., 2023; Reggio et al., 2020b; Trensz et al., 2010). Canonical WNT3A signaling promoted the conversion of FAPs into Collagen-secreting fibroblasts (Trensz et al., 2010). Conversely, the non-canonical WNT7A has been demonstrated capable of abrogating FAP adipogenic tendencies by activating WNT–RHO–YAP/TAZ signaling (Fu et al., 2023).

In addition, our group demonstrated that the reconstitution of a functional WNT5A–GSK3–β-catenin axis restricts adipogenesis in dystrophic FAPs (Reggio et al., 2020b). Remarkably, we demonstrated that WNT-phenocopying agents, namely GSK3 inhibitors, can protect muscles from glycerol-induced fatty degeneration (Reggio et al., 2020b). Therefore, these and other studies imply that, despite certain challenges, ad hoc manipulation of WNT pathway components is eligible for designing novel therapies to selectively target FAP adipogenicity.

To increase the clinical value of these observations, in the present work, we identified the β-catenin transcriptional complex as an entity that intimately represses adipogenesis in adipose precursor cells. Here, we tested the potential use of the GSK3 inhibitor LY2090314 as a WNT-phenocopying agent that stimulates stabilization and accumulation of β-catenin. LY2090314 is a highly potent GSK3 inhibitor, exerting its anti-adipogenic effect in the nanomolar range, significantly reducing off-target effects.

We demonstrated that the anti-adipogenic effect of LY2090314 is conserved in a panel of murine and human adipose progenitor cells, including hFAPs. This suggests that β-catenin-mediated adipogenic repression is a highly conserved mechanism that can be manipulated to control the differentiation of adipose precursor cells. Moreover, using LC-MS/MS profiling, we uncovered that LY2090314 exerts WNT-mimicking effects, inhibiting hFAP adipogenesis through, at least, two independent mechanisms, both mediated by β-catenin: a primary mechanism ascribed to the role of LY2090314 in inhibiting GSK kinase, and a second indirect mechanism that corroborates GSK blockage via upregulation of WNT5A.

Translating promising data in successful pre-clinical studies and/or clinical trials remains one of the main challenges for research that is focused on identifying novel pharmacological compounds for treatment of dystrophies. Indeed, although promising, most of these data are limited to basic *in vitro* systems (i.e. 2D cultures) and have yet to be verified in humanized animal models. Such drawbacks are often caused by limited testing steps in most complex culture scenarios, for which it would be necessary to adjust doses, evaluate cellular responses and model the clinical outcomes at cell-resolution level. To overcome such limitation, we presented the possibility of generating 3D models of fat infiltrates by combining the photopolymerizable PF biomimetic matrix with mFAPs and hFAPs. In this 3D state, FAPs are viable and graft in host recipient mice. Most importantly, in this 3D system, FAPs maintained their ability to respond to external stimuli and differentiate into adipocytes. Moreover, one of the great advantages of this ectopic surrogate is the possibility to plan long-term culturing/live monitoring of these cells, allowing longitudinal analyses and tests. By exploiting this system, we confirmed that a single two-digit nanomolar dose of LY2090314 is sufficient to abrogate 3D FAP adipogenesis, concretizing the possibility to use LY2090314 to limit fatty degeneration in higher systems. The integration of our current molecular knowledge on FAPs with the development of 3D humanized disease models will help in the development of clinical therapeutic interventions for treating secondary complications of human dystrophy.

## MATERIALS AND METHODS

### Mouse models

C57BL/6J and NOD/SCID mice were bred and maintained according to standard facility procedures. Experiments on animals were conducted according to the rules of good animal experimentation (IACUC No. 432 of 12 March 2006) and under Italian Health Ministry Approval No. 271/2021-PR.

### Human samples

The studies involving human participants were reviewed and approved by the Istituti Fisioterapici Ospitalieri (IFO) ethics committee. The participants provided their written informed consent, in line with the Declaration of Helsinki. All clinical investigation was conducted according to the principles expressed in the Declaration of Helsinki. Donors (XX53, XY65 and XX57) were all healthy. Biopsies were taken during orthopedical intervention, and FAPs were isolated from muscle biopsies. hASCs were isolated from subcutaneous fat tissue.

### Isolation of primary mFAPs

mFAPs were isolated from the hind limbs of male wild-type C57BL/6J mice. Briefly, hind limbs were surgically removed and then minced in Hanks’ balanced salt solution (HBSS; Gibco) supplemented with 100 U/ml penicillin/streptomycin (P/S; Roche) and 0.2% bovine serum albumin (BSA; AppliChem). For each mouse, the homogeneous muscle tissue preparation was enzymatically digested in 2 μg/μl collagenase A, 2.4 U/ml dispase II, and 10 μg/ml DNase I (Roche) in Dulbecco's phosphate-buffered saline (BioWest) with calcium and magnesium. Enzymatic digestion was performed for 1 h at 37°C with gentle shaking. The homogenate underwent consecutive filtration through 100, 70 and 40 μm cell strainers (Corning). Before each filtration step, cells were centrifuged at 700 ***g*** for 10 min at 4°C and then resuspended in fresh HBSS. Red blood cells were lysed in RBC lysis buffer (Santa Cruz Biotechnology). Freshly isolated muscle mononuclear cells were then resuspended in magnetic bead buffer (0.5% BSA and 2 mM EDTA in 1× PBS) and filtered through a 30 μm Pre-Separation Filter (Miltenyi) to remove large particles. The whole-cell suspension underwent subsequential incubations with the microbead-conjugated antibodies used for magnetic sorting. The sorting procedures and labeling procedures with the microbead-conjugated antibodies were performed according to the manufacturer's instructions. FAPs were selected as LIN^−^/ITGA7^−^/Sca-1^+^ (ATXN1^+^) cells.

Freshly sorted mFAPs were resuspended in FAP-GM consisting of high-glucose (25 mM) DMEM GlutaMAX (Gibco) supplemented with 20% FBS, 10 mM Hepes, 1 mM sodium pyruvate and 100 U/ml P/S. After 4 days the FAP-GM was fully refreshed, and cells were cultured for two additional days before the induction of adipogenic differentiation. Adipogenic differentiation was induced by incubating FAPs with the ADM (FAP-GM supplemented with 1 μg/ml insulin, 0.5 mM 3-isobutyl-1-methylxanthine and 1 μM dexamethasone) for 3 days followed by two additional days in adipocyte maintenance medium (AMM; FAP-GM supplemented with 1 μg/ml insulin). Unstimulated cells were maintained in fresh FAP-GM.

### Isolation of primary hFAPs

Muscle biopsies were obtained as res nullius from surgeries on healthy donors, thanks to a collaboration with the IFO. Muscle homogenates were subjected to the same protocol used to release mFAPs, in the presence of dispase, collagenase and DNase I. The resulting suspension was filtered and plated for 10 days in Cytogrow (Resnova) to allow cell amplification. For enriching hFAPs, amplified cells were stained with microbead-conjugated antibodies and selected as CD56^−^/CD15^+^ cells. Freshly sorted hFAPs were cultured and differentiated into adipocytes by adopting the protocols tested for the murine counterparts. Notably, to enhance adipogenesis of these cells, 1 µM rosiglitazone was added to standard ADM.

### Generation of a 3D model of fatty infiltration

All the experiments were performed using PF biomimetic matrix. 3D fat infiltrate models were fabricated by a casting method. Briefly, 8 mg/ml PF, supplemented with 0.1% Irgacure™ 2959 photoinitiator solution (Ciba Specialty Chemicals), was loaded with hFAPs or mFAPs. The fork was placed in the mold, and the cell-hydrogel mixture was poured into a PTFE caster. The mixture was polymerized by exposing the mold chamber to non-toxic and low-penetrating UV light, for 5 min. The polymerized constructs were gently removed and transferred to normal dishes containing FAP-GM and then used according to experimental need.

### Transplantation of 3D constructs loaded with hFAPs

Two-month-old male NOD/SCID mice (*n*=5) were anesthetized with a 1:1 mixture of ketamine (5 mg/ml; MSD Animal Health, Ketavet 100) and xylazine (1 mg/ml; Rompun Bayer) at a dose of 10 ml/kg intramuscularly. A limited skin incision on the medial side of the back was introduced, dorsal muscle was separated from the skin, the hFAP-derived construct was carefully positioned, and skin closure was performed by non-absorbable 6-0 silk sutures (Clinsilk). Mice were sacrificed 28 days after implantation for morphological analysis.

### Isolation of primary hASCs

Fat biopsies were obtained as res nullius from surgeries on healthy donors, thanks to a collaboration with the IFO. To release hASCs, fat biopsies were digested with an enzymatic mix containing collagenase type II for 1 h at 37°C. hASCs were cultured and differentiated using the same protocols adopted for hFAPs.

### Drug compounds

All the compounds used in this study were purchased from Selleckchem and reconstituted according to the manufacturer's instructions.

### Glycerol injury and muscle section preparation and labeling

For glycerol muscle injury 50 μl of 50% v/v hypertonic solution of glycerol was administered intramuscularly into the tibialis anterior of C57BL/6J mice. The contralateral limb was injected with an equal volume of saline solution, as an internal control. After 14 days, mice were sacrificed, and the hind limb muscles were surgically removed, embedded in optimal cutting temperature compound and snap-frozen in liquid nitrogen. Muscle sections of 10 µm thickness were obtained using a Leica cryostat. Muscle sections were immunolabeled for PDGFRα and Perilipin expression.

### Real-time PCR

Total RNA was extracted using TRIzol. Before resuspension, total RNA was precipitated overnight in the presence of 10 μg glycogen. RNA concentration was assessed using a NanoDrop Lite Spectrophotometer (Thermo Fisher Scientific). Total RNA (1000 ng) was reverse transcribed into cDNA with a PrimeScript RT Reagent Kit (TakaraBio, RR037A). Quantitative PCR reactions were carried out with SYBR Premix Ex Taq (Tli RNaseH Plus; TakaraBio, RR420) and performed in technical duplicates for each biological repeat. Each reaction mixture (final volume of 20 μl) contained 50 ng cDNA. *Actb* was used as reference gene.

### Immunoblotting

Cells were washed in 1× PBS and lysed in ice-cold lysis buffer (150 mM NaCl, 50 mM Tris-HCl pH 7.5, 1% Nonidet P-40, 1 mM EDTA, 1% Triton X-100) supplemented with 1 mM ortovanadate, 1 mM NaF, protease inhibitor mixture 1× (Sigma-Aldrich, P8340), inhibitor phosphatase mixture II 1× (Sigma-Aldrich, 5726) and inhibitor phosphatase mixture III 1× (Sigma-Aldrich, P0044) prior to use. Cell lysates were incubated on ice for 30 min and then separated at 15,500 ***g*** in a refrigerated centrifuge. Protein concentration was estimated using Bradford reagent (Bio-Rad, 500-0006). Total protein extracts were resolved by 10%, 15% SDS-PAGE or 4-20% Bio-Rad CRITERION^®^ gradient gel according to need. Proteins were transferred to Trans-Blot^®^ Turbo mini or midi nitrocellulose membranes (Bio-Rad, 1704156–1704157) using a Trans-Blot^®^ Turbo™ transfer System (Bio-Rad), and the non-specific sticky sites on the membranes were saturated for 1 h at room temperature in blocking solution (5% milk, 0.1% Tween 20 in 1× Tris-buffered saline). Saturated membranes were incubated with specific primary antibodies diluted in blocking solution according to the manufacturer's instructions. The binding of primary antibodies was revealed using host-specific secondary antibodies. Chemiluminescent detection was performed using Clarity™ Western ECL Blotting Substrates (Bio-Rad, 1705061) and a Fujifilm Las-3000 imaging system. Band densities were quantified using ImageJ. The brightness and contrast of each blot was adjusted using the ‘Auto Contrast’ function in Adobe Photoshop. The full list of antibodies used is provided in Table S5.

### ORO

ORO (Sigma-Aldrich, O0625) stock solution was prepared according to the manufacturer's instructions. Fixed cells were washed with 1× PBS and incubated for 10 min with filtered ORO working solution (3:2 ratio, ORO:ultrapure water). Stained cells were washed twice for 10 min with 1× PBS and counterstained using Hoechst 33342 (Thermo Fisher Scientific, 62249). ORO-stained cells were acquired via fluorescence or brightfield microscopy.

### Image acquisition and analysis

Images of immunolabeled cells and sections were acquired using a DMI6000B fluorescent microscope (Leica). Images were scored manually using Fiji by two independent collaborators that were unaware of the treatment condition. Results are expressed as ratio of the total objects counted.

### Proteome sample preparation

Cells were harvested as indicated in the text and directly lysed in ice-cold RIPA buffer. Proteome preparation was done using the in-StageTip (iST) method (Kulak et al., 2014; Reggio et al., 2021). Samples were separated by high-performance liquid chromatography in a single run (without pre-fractionations) and analyzed by LC-MS/MS.

### LC-MS/MS measurements

Instruments for LC-MS/MS analysis consisted of a NanoLC 1200 (Thermo Fisher Scientific) coupled via a nano-electrospray ionization source to a quadrupole-based Q Exactive HF benchtop mass spectrometer (Thermo Fisher Scientific). Peptide separation was carried out according to hydrophobicity on a home-made column [75 μm inner diameter, 8 μm tip, 400 mm bed packed with Reprosil-PUR, C18-AQ, 1.9 μm particle size, 120 Å pore size (New Objective, PF7508-250H363)], using a binary buffer system consisting of solution A (0.1% formic acid) and B (80% acetonitrile, 0.1% formic acid). Total flow rate was 300 nl/min. For the liquid chromatography linear gradient, after sample loading, the run started at 5% buffer B for 5 min, followed by a series of linear gradients, from 5% to 30% B in 90 min, then a 10 min step to reach 50% and a 5 min step to reach 95%. This last step was maintained for 10 min.

MS spectra were acquired using 3×10^6^ as an automatic gain control (AGC) target, a maximal injection time of 20 ms and 120,000 resolution at 200 m/z. The mass spectrometer operated in data-dependent Top20 mode with subsequent acquisition of higher-energy collisional dissociation fragmentation MS/MS spectra of the top 20 most intense peaks. Resolution for MS/MS spectra was set to 15,000 at 200 m/z, AGC target to 1×10^5^, maximum injection time to 20 ms and the isolation window to 1.6 Th. The intensity threshold was set at 2.0×10^4^ and dynamic exclusion at 30 s.

### Proteome data processing

All acquired raw files were processed using MaxQuant (1.6.2.10) and the implemented Andromeda search engine. For protein assignment, spectra were correlated with the Human (v. 2021) reference proteome, including a list of common contaminants. Searches were performed with tryptic specifications and default settings for mass tolerances for MS and MS/MS spectra. The other parameters were set as follows: fixed modification, carbamidomethyl (C); variable modifications, oxidation, acetyl (N-term); digestion, trypsin, Lys-C; minimum peptide length, 7; maximum peptide mass, 470 Da; false discovery rate for proteins and peptide spectrum, 1%.

For further analysis, Perseus software (1.6.2.3) (Tyanova et al., 2016) was used, and first filtered for contaminants and reverse entries as well as proteins that were only identified by a modified peptide (first filter). The label-free quantitation (LFQ) ratios were logarithmized, grouped and filtered for minimum valid number (minimum of three in at least one group) (second filter).

Missing values were replaced by random numbers drawn from a normal distribution. Two-sample *t*-test analysis was performed using FDR=0.05. Proteins with Log_2_ difference ≥±1 and *q*-value <0.01 were considered significantly enriched.

Categorical annotation was added in Perseus in the form of GO biological process, GO molecular function and GO cellular component, and Kyoto Encyclopedia of Genes and Genomes (KEGG) pathways.

### Protein network generation

This strategy has been previously developed and applied to query complex proteome datasets (Reggio et al., 2020a; Sacco et al., 2019). Casual relationships between significant protein entities (coming from the LY2090314-versus-vehicle comparison) were retrieved from the SIGNOR database (Lo Surdo et al., 2023) using a dedicated application in the Cytoscape platform (Shannon et al., 2003). Nodes were color coded according to their difference value (LY2090314-versus-vehicle comparison); the dimension of nodes is proportional to their *q*-value.

## Supplementary Material

10.1242/dmm.049915_sup1 Supplementary information
