# Supplementary material for: A 3D adipogenesis platform to study the fate of fibro/adipogenic progenitors in muscular dystrophies
Source: Dis Model Mech. 2023 Jun 23;16(6):dmm049915. doi: 10.1242/dmm.049915 (PMC10309591; doi:10.1242/dmm.049915)
Supplement: Supplementary information [file dmm-16-049915-s1.pdf]

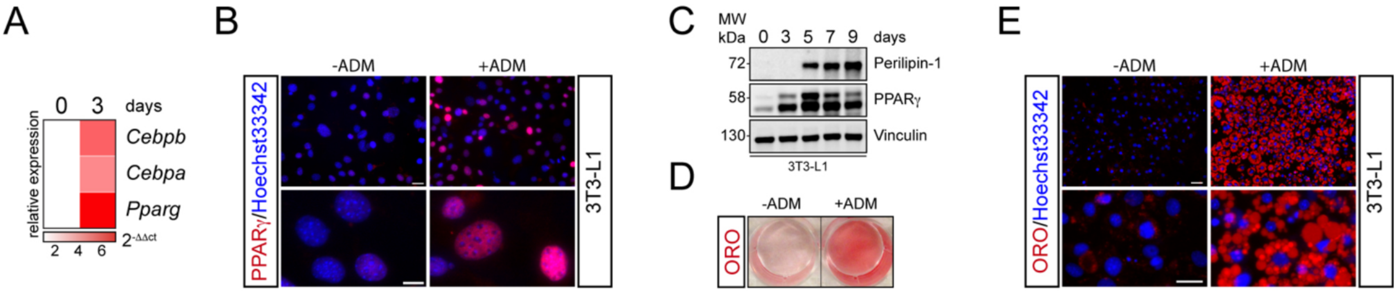

Fig. S1. 3T3-L1 cells undergo adipogenesis upon ADM stimulation. (A) Heat map showing the differential gene expression of Cebpb, Cebpa and Pparg, between unstimulated and ADM-induced 3T3-L1 cells. (B) Representative immunofluorescence showing PPARγ expression and localization in 3T3-L1 cells cultured either in growth medium or in ADM for three days. (C) Representative western blot showing perilipin-1 and PPARγ expression in 3T3-L1 undergoing adipogenic differentiation. Vinculin serves as loading control. (D) Representative ORO staining in 3T3-L1 cells in the presence of vehicle or ADM. (E) Representative immunofluorescence showing ORO staining in 3T3-L1 cells stimulated with the vehicle or ADM. Nuclei (blue) were labelled with Hoechst 33342. All micrographs were captured at 20× magnification. Scale bar: 50 μ m; Scale bars of insets: 10 μm. Figures and data are representative of at least three independent biological repeats.

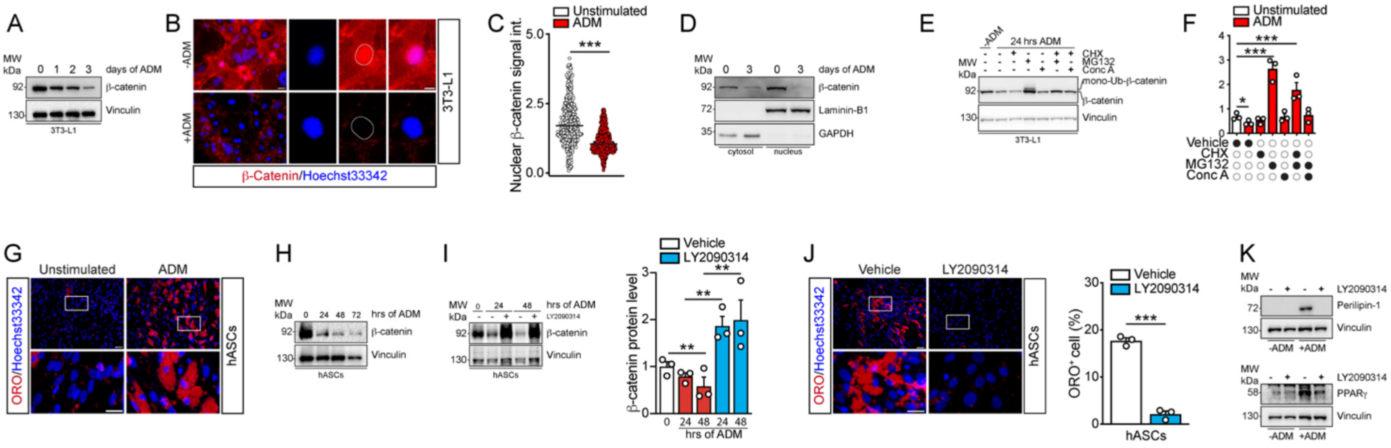

Fig. S2. β-Cateinin degradation hallmarks adipogenesis. (A) Representative western blot showing β-catenin expression in 3T3-L1 during the first three days of differentiation. Vinculin serves as loading control. (B) Representative immunofluorescence showing β-catenin expression and localization in 3T3-L1 cells cultured either in growth medium or in ADM for three days. Nuclei (blue) were labelled with Hoechst 33342. (C) Dot plot showing the relative quantification of the nuclear β-catenin signal in 3T3-L1 cells cultured either in growth medium or in ADM for three days. (D) Representative western blot showing β-catenin expression in cytoplasmic and nuclear enriched fractions collected from 3T3-L1 undergoing adipogenesis. (E) Representative western blot showing β-catenin expression in 3T3-L1 exposed to cycloheximide, MG132 and Concanamycin A, after 24 hours of ADM. Vinculin serves as loading control. (F) Bar plot reporting the relative quantification of β-catenin expression levels.

Disease Models & Mechanisms • Supplementary information

(G) Representative immunofluorescence showing ORO staining in hASCs exposed or not to ADM. Nuclei (blue) were labelled with Hoechst 33342. (H) Representative western blot showing β-catenin expression in hASCs during the first three days of differentiation. Vinculin serves as loading control. (I) Representative western blot showing the expression of β- catenin proteins during the first three days of differentiation of hASCs. Differentiating cells were incubated in the presence/absence of 20 nM of LY2090314. Vinculin serves as loading control. Associated bar plot reporting the relative quantification of the β-catenin expression. (J) Representative immunofluorescence showing ORO staining in hASCs exposed or not to 20 nM of LY2090314. Nuclei (blue) were labelled with Hoechst 33342. Relative bar plot reporting the fraction of ORO-positive cells in both conditions. (K) Representative immunoblots showing perilipin-1 and PPARγ expression in hASCs differentiated in the presence/absence of 20 nM of LY2090314. Vinculin serves as loading control. All micrographs were captured at 20× magnification. Scale bar: 50 μm; Scale bars of insets: 10 μm. Figures and data are representative of at least three independent biological repeats.

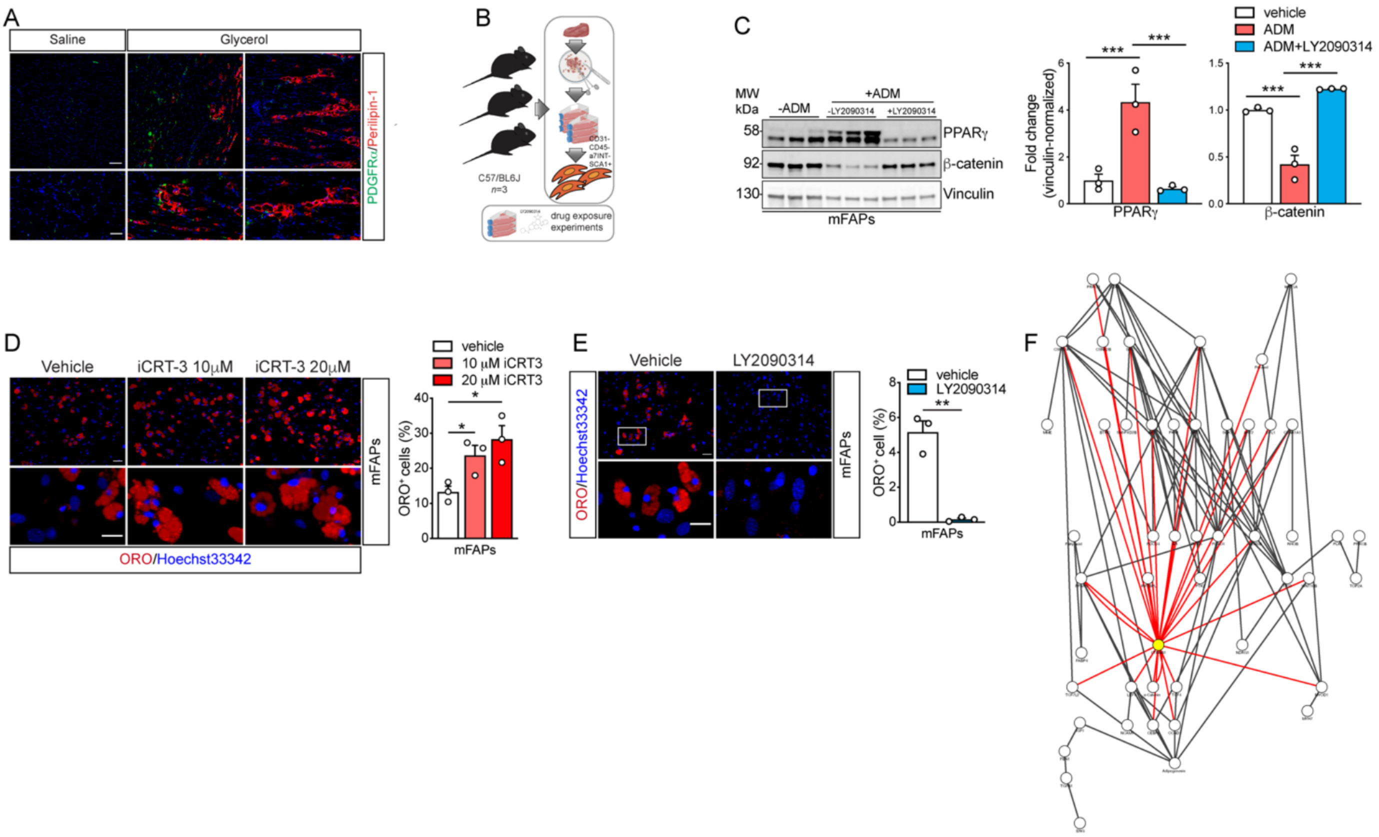

Fig. S3. LY2090314 halts adipogenesis of adipose progenitor cells. (A) Representative immunofluorescence showing tibialis anterior muscle sections stained for the expression of perilipin-1 and PDGFRa. Tibialis anterior muscles were either injected with saline solution or with 50 ml of 50% v/v of glycerol solution. Muscles were sampled after 14 days. (B) Representative scheme showing collection and utilization of murine- derived FAPs (mFAPs). (C) Representative western blot showing the expression of b-catenin and PPARg proteins during the first three days of differentiation of 3T3-L1, in the absence/presence of 20 nM LY2090314. Vinculin is used as loading control. The bar plot reports the densitometric values of b-catenin in both conditions. (D) Representative immunofluorescence analysis showing ORO staining in differentiated mFAPs after the exposure to increasing doses of iCRT-3 (10 and 20 μM). Nuclei (blue) were revealed using Hoechst 33342. (E) Representative ORO staining showing differentiated murine FAPs (mFAPs) in the presence/ absence of 20nM of LY2090314. Nuclei (blue) were revealed using Hoechst 33342. The associated bar plot reports the percentage of ORO-positive cells in both conditions. (F) Network showing the connectivity between significant and “bridge” proteins to adipogenesis. CTNNB1 is highlighted in yellow while incoming and outcoming stimuli are reported in red.

Table S1. Full table reporting proteins profiled via high-resolution mass spectrometry.

Click here to download Table S1

Table S2. List of GO terms that are positively enriched in the LY2090314-vs-vehicle comparison.

Click here to download Table S2

Table S3. List of GO terms that are negatively enriched in the LY2090314-vs-vehicle comparison.

Click here to download Table S3

Table S4. Casual interactions between protein entities that are part of LY2090314-specific network.

Click here to download Table S4

Table S5. Reagents used in this study.

Click here to download Table S5

Disease Models & Mechanisms • Supplementary information
